# The Complete Mitochondrial Genome of *Entemnotrochus rumphii*, a Living Fossil for Vetigastropoda (Mollusca: Gastropoda)

**DOI:** 10.3390/genes13112061

**Published:** 2022-11-07

**Authors:** Yunan Wang, Peizhen Ma, Zhen Zhang, Cui Li, Yumeng Liu, Ya Chen, Jiahui Wang, Haiyan Wang, Hao Song

**Affiliations:** 1 Institute of Oceanology, Chinese Academy of Sciences, No. 7 Nanhai Road, Qingdao 266071, China; 2 Laboratory for Marine Ecology and Environmental Science & Marine Biology and Biotechnology, Qingdao National Laboratory for Marine Science and Technology, Qingdao, 266237, China; 3 College of Earth and Planetary Sciences, University of Chinese Academy of Sciences, Beijing 101400, China; 4 Center for Ocean Mega-Science, Chinese Academy of Sciences, Qingdao 266071, China

**Keywords:** *Entemnotrochus rumphii*, mitochondrial genome, phylogenetic analysis, Vetigastropoda

## Abstract

Pleurotomarioidea represents a truly isolated and basally diverging lineage in Vetigastropoda (Mollusca: Gastropoda) whose fossil record can date back to the late Cambrian, thus providing rare insights into the evolutionary history of molluscs. Here, we sequenced and assembled the complete mitochondrial genome of one representative species from Pleurotomarioidea—*Entemnotrochus rumphii* (Schepman, 1879)—of which the mitogenome is 15,795 bp in length, including 13 protein-coding genes, two ribosomal RNA genes, and 22 transfer RNA genes. The nucleotide composition was biased toward AT, and A + T content reached 65.2%. *E. rumphii* was recovered as sister to all other living vetigastropods according to mitogenome-based phylogenetic analysis. The mitochondrial gene order was consistent with major vetigastropods and the hypothetical ancestral gastropoda, suggesting the deep conservation of mitogenome arrangement in Vetigastropoda.

## 1. Introduction

*Entemnotrochus rumphii* (Schepman, 1879) belongs to the superfamily Pleurotomarioidea, subclass Vetigastropoda, which is regarded as one of the most deeply diverging lineages among Gastropoda [1,2]. Pleurotomarioids can be distinguished from other gastropods based on their special morphological characteristics such as the coiled, conispiral shells and a slit along outer lips [3]. Pleurotomarioids are distributed in tropical and temperate latitudes [4]. Two species of *Entemnotrochus* are widely distributed, and one is *E. rumphii*, which is known from the southern islands of Japan to the central Philippines and Indonesia [3]. According to museum records, the bathymetric ranges of living *E. rumphii* range from about 80–280 m, and they inhabit sandy, coarse bottoms [5]. Tan [6] reported that the gut content and feces of *E. rumphii* consisted of sponge spicules, which indicated that this species lived on a diet of sponges.

Pleurotomarioidea is the only Gastropoda superfamily with fossil records from the Cambrian to the present and they are called “living fossils” [7,8,9,10]. The family Pleurotomariidae originated in the lower Triassic and is the only family of Pleurotomarioidea that survives now. Of 1500 described species of Pleurotomarioidea, only 25 are living today [4]. Their symmetrical organs and asymmetrical shells are regarded as transitional between symmetrical ancestors and asymmetrical modern gastropods [11,12]. Overall, these species have significance for understanding the early evolution of Gastropoda.

The mitochondrion is an important organelle in eukaryotes, and the mitochondrial genome has nearly the same structure in all animals, that is, a circular double-stranded DNA molecule, almost always including 13 protein-coding genes (PCGs), two ribosomal RNAs, and 22 transfer RNAs [13]. The conservation of its composition and structure makes the mitochondrial genome a good material for studying evolution and phylogenetic relationships [14,15]. Even as mitochondrial genome data are increasing for Gastropoda, few have been published yet for Vetigastropoda, especially within Pleurotomariida. To provide more molecular information on this order, we described a complete mitochondrial genome of *E. rumphii*, a representative species of Pleurotomarioidea. We also conducted a phylogenetic analysis and compared gene arrangements within Vetigastropoda.

## 2. Materials and Methods

### 2.1. Sample Collection and DNA Extraction

A specimen of *E. rumphii* was collected in 2020 from the East China Sea, near Japan (30.9° N, 129.9° E). The entire individual was preserved at −80 °C in the laboratory at the Institute of Oceanology, Chinese Academy of Sciences, Qingdao, China. Total genomic DNA was extracted from its foot using CTAB methods.

### 2.2. Mitogenome Sequencing and Assembly

The complete mitochondrial genome was sequenced and assembled by Huitong Biotechnology, Shenzhen. Genomic libraries with an average length of 350 bp were constructed using the NexteraXT DNA Library Preparation Kit and then sequenced as 150 bp paired-end runs on the Illumina Novaseq platform (Total Genomics Solution Limited, SZHT) (San Diego, CA, USA). Raw data were filtered using the NGS QC Toolkit v2.3.3 [16] for quality control. De novo assembling was performed with SPAdes 3.11.0 [17].

### 2.3. Mitogenome Annotation, Sequence Analysis, and Data Acquisition

The mitochondrial genome of *E. rumphii* was annotated using MITOS Web Server (http://mitos.bioinf.uni-leipzig.de/index.py, accessed on 13 July 2021). Manual corrections of genes for start/stop codons were performed in SnapGene Viewer. The position and size of all protein-coding genes were corrected using Open Reading Frame Finder (ORFfinder Home–NCBI (nih.gov)). Finally, a mitochondrial genome map was visualized with the online tool Proksee (https://proksee.ca/, accessed on 3 August 2022). The nucleotide composition, composition skew, codon usage of PCGs, and relative synonymous codon usage (RSCU) were analyzed using PhyloSuite v1.2.2 [18]. The secondary structures of 22 tRNAs were predicted using tRNAscan-Se [19,20] and MITOS, and drawn with the ViennaRNA Web Services (http://rna.tbi.univie.ac.at/, accessed on 21 September 2022).

To explore the phylogenetic relationship and gene arrangement of Vetigastropoda, 21 complete mitochondrial genomes were obtained from the NCBI database (Table 1). Of these, 19 species belong to the subclass Vetigastropoda, while the other two species from the subclass Neritimorpha were viewed as outgroups.

### 2.4. Phylogenetic Analyses

To indicate the phylogenetic position of *E. rumphii* among Vetigastropoda, phylogenetic analysis was performed. Thirteen protein-coding genes were obtained using PhyloSuite v1.2.2 [18]. Amino acid sequences were aligned with Mafft v7 [21], and the misaligned positions were removed using Gblocks 0.91b [22] with default settings. All PCGs were concatenated using PhyloSuite v1.2.2 [18]. The best-fitting partition model was found by ModelFinder [23] using the Corrected Akaike Information Criterion (AICc). A maximum-likelihood (ML) tree was reconstructed by IQ-Tree v2.1.2 [24] with 1000 ultrafast bootstrap replications. Finally, the phylogenetic tree was shown by FigTree v1.4.4.

## 3. Results and Discussion

### 3.1. Mitogenome Characteristic

**Figure 1:**
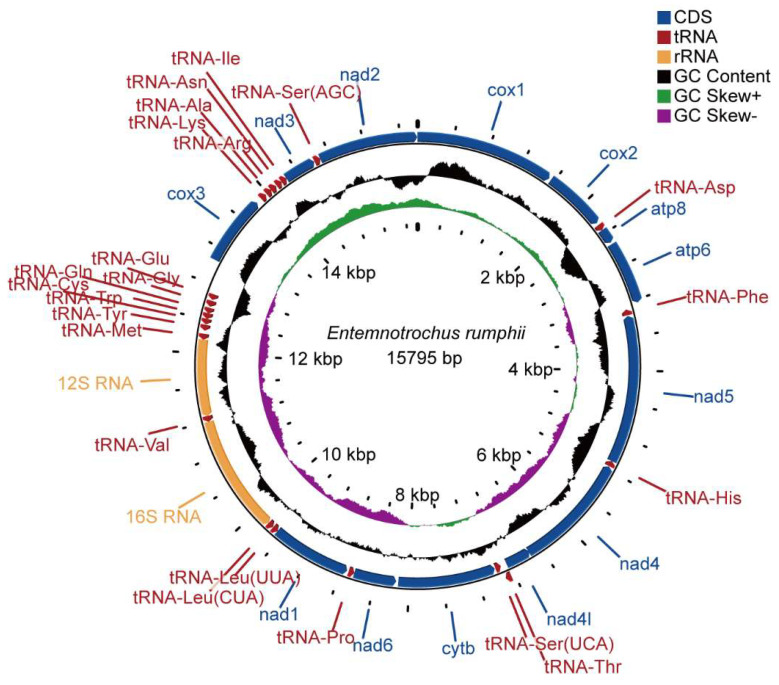
The mitochondrial genome map of *Entemnotrochus. rumphii*. From outside to inside, the first circle represents gene arrangement, the second represents the GC content, the third represents the GC skew, and the innermost circle represents the scale. The GC content circus was centered at 50% and the GC skew was centered at zero.

The circular mitogenome of *E. rumphii* was 15,795 bp in length, containing 13 protein-coding genes (PCGs), two ribosomal RNA genes (*rrnS* and *rrnL*), and 22 transfer RNA genes (Figure 1; Table 2). The length of this mitogenome was smaller than other vetigastropods except for *Lepetodrilus schrolli* (Table 3). Seven PCGs (*cox1*, *cox2*, *cox3*, *nad2*, *nad3*, *atp6*, *atp8*) and eight tRNAs (*tRNA-Ser^AGC^*, *tRNA-Ile*, *tRNA-Asn*, *tRNA-Arg*, *tRNA-Ala*, *tRNA-Lys*, *tRNA-Thr*, *tRNA-Asp*) were encoded on the heavy strand, while the other genes, including two ribosomal RNAs, were encoded on the light strand. Twenty-two intergenic regions were found ranging from 1 to 364 bp, while the largest one was located between *tRNA-Glu* and *cox3*. Moreover, two overlaps were detected. *Nad4* overlapped *nad4l* by seven bp, and *tRNA-Thr* overlapped *tRNA-Ser^UCA^* by one bp.

The nucleotide composition was 35.21% for A, 29.98% for T, 20.39% for C, and 14.43% for G, indicating it was biased toward A+T content at 65.19%, which was similar to other vetigastropods (Table 3). The AT skew of *E. rumphii* was positive, while the GC skew was negative. Among 20 vetigastropods analyzed in our study, the AT skews of eight species were positive, and the GC skews of 13 species were negative, not showing any apparent consistency.

### 3.2. Protein-Coding Genes

The total length of all PCGs was 11,310 bp, accounting for 71.6% of the complete mitochondrial genome. The size of the 13 PCGs ranged from 165 (*atp8*) to 1731 bp (*nad5*). All PCGs used ATG as the start codon. Among the 20 vetigastropods we studied, ATG was the most frequently used start codon and 11 species used it for all PCGs. Two different kinds of stop codons were found in the 13 PCGs; TAA in nine PCGs (*cox1*, *cox2*, *cox3*, *cytb*, *nad2*, *nad3*, *nad4*, *nad5*, *atp8*) and TAG in the remaining four PCGs (*nad1*, *nad4l*, *nad6*, *atp6*). The other 19 vetigastropods also used TAA and TAG as stop codons.

The base composition of the PCGs was 25.2% for A, 40% for T, 15.4% for C, and 19.4% for G. The A + T content of each individual PCG and the concatenated data all exceeded 60%, which showed that the PCGs preferred AT. Except for *atp8*, all PCGs had a negative AT skew, suggesting they were biased toward T. As for GC skew, eight PCGs had a positive GC skew, while the other five PCGs had a negative one.

**Figure 2:**
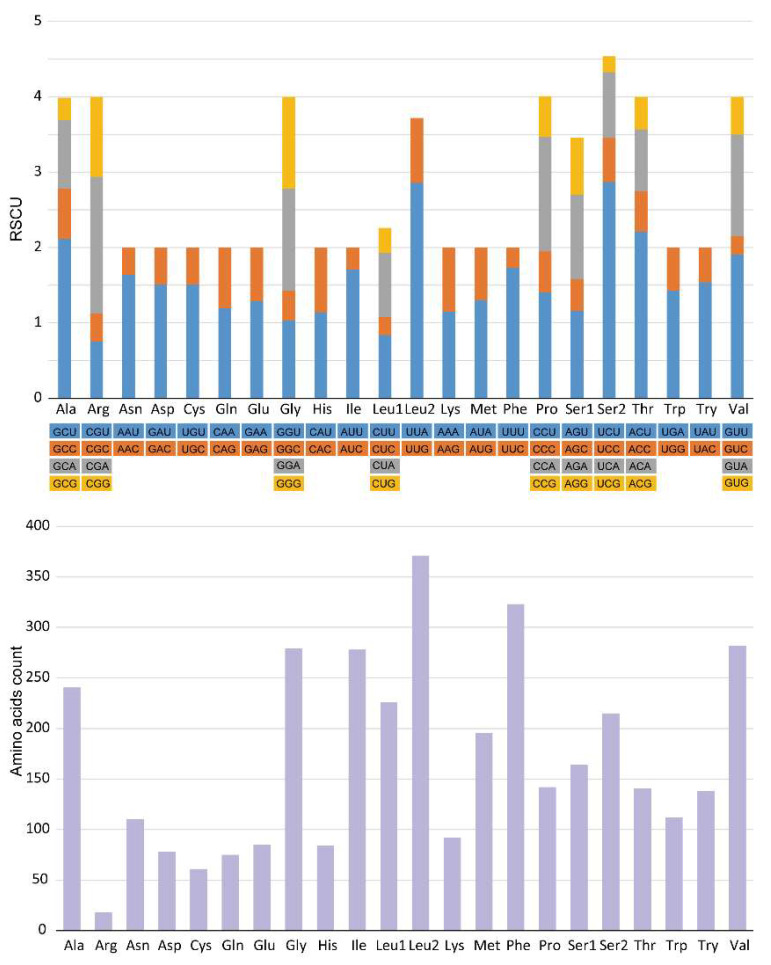
Relative synonymous codon usage (RSCU) and amino acids count of *E. rumphii* mitochondrial genome.

The relative synonymous codon usage (RSCU) of *E. rumphii* mitogenome was presented in Figure 2, indicating Leu, Phe, and Val were the three most frequently used amino acids. UUA-Leu, UUU-Phe, and AUU-Ile were the three most frequently utilized codons. Among 22 amino acids, nine (Ala, Arg, Gly, Leu1, Pro, Ser1, Ser2, Thr, and Val) had four codons, and others had two codons.

### 3.3. Transfer RNAs and Ribosomal RNAs

**Figure 3:**
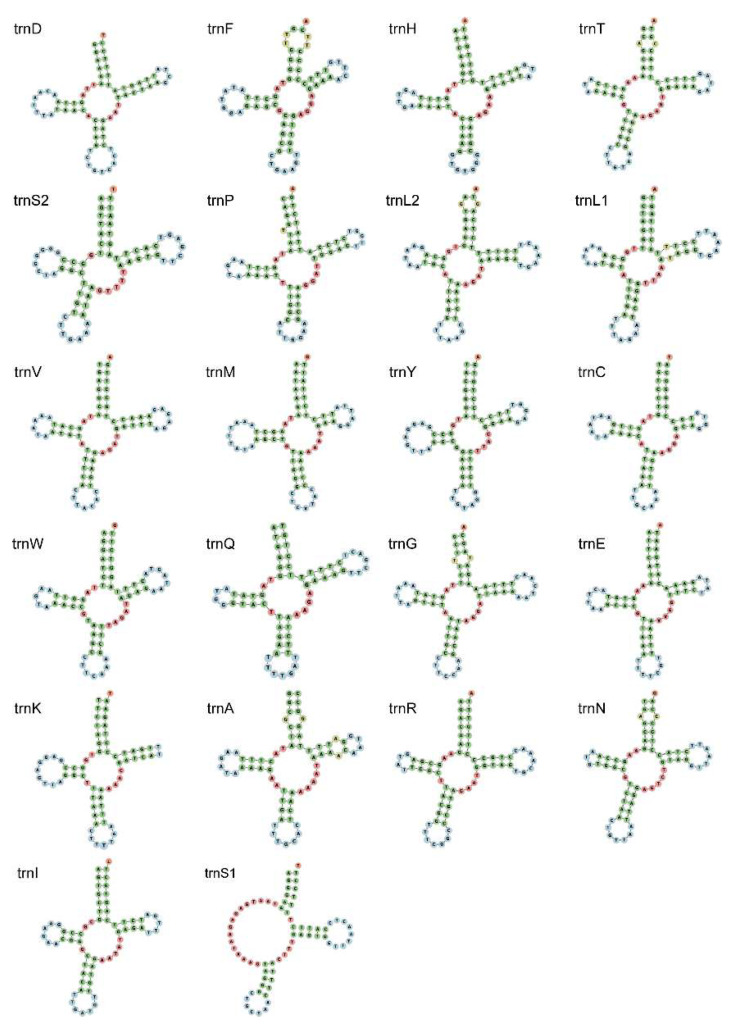
Secondary structure of 22 tRNAs of *E. rumphii* mitochondrial genome.

The size of 22 transfer RNA genes ranged from 64 (*tRNA-Cys*) to 70 bp (*tRNA-Gly*). The AT content of total tRNAs was 62.6%, also showing an AT bias. Almost all tRNAs of *E. rumphii* had typical clover-leaf secondary structures with four arms, except for *trnS1* (Figure 3). This phenomenon where this specific tRNA does not form a four-arms clover-leaf structure can be observed in most metazoan mitogenomes [25].

The 12S ribosomal RNA (*rrnS*) had a length of 894 bp, and the 16S ribosomal RNA (*rrnL*) had a length of 1386 bp. The 12S ribosomal RNA was located between *tRNA-Val* and *tRNA-Met*, while the 16S ribosomal RNA gene was located between *tRNA-Leu^CUA^* and *tRNA-Val*. For *rrnL*, the AT skew and GC skew values were −0.118 and 0.321; for *rrnS*, they were −0.054 and 0.321. Both of these rRNAs were biased toward T and G.

### 3.4. Phylogenetic Analysis

**Figure 4:**
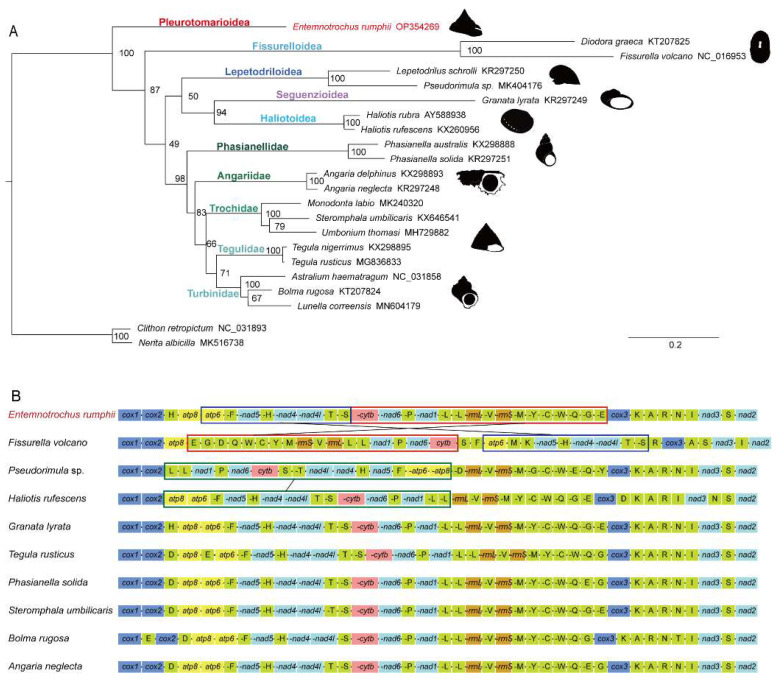
Phylogenetic tree of *Entemnotrochus rumphii*, 20 species of Vetigastropoda, and 2 outgroup species based on the amino acid sequences of 13 protein-coding genes (**A**), and protein-coding gene arrangement of 10 species in Vetigastropoda (**B**). In the phylogenetic tree, bootstrap support values (BS) are shown on each node. The arrangement graph is shown with iTOL (https://itol.embl.de/, accessed on 15 October 2022) and the boxes in the same color show identical gene blocks.

In our study, phylogenetic analysis of Vetigastropoda was conducted based on 13 protein-coding genes (Figure 4A). Based on two Neritimorpha species *Clithon retropictus* and *Nerita albicilla* as outgroups, the vetigastropods grouped together in a single clade. Within Vetigastropoda, *E. rumphii* was recovered as sister to all other vetigastropods (BS = 100). Other vetigastropods were divided into three major clades. *Fissurella volcano* and *Diodora graeca*, representing Fissurelloidea, formed the first clade with full support. Lepetodriloidea, Haliotidea, and Seguenzioidea formed the second clade, though without significant support (BS = 50). Five families of Trochidea formed the third clade (BS = 98). However, the relationship of these clades remained unresolved because all bootstrap support values at deep nodes were lower than 95. Among Trochidea, all families were recovered as monophyletic groups with full support. Phasianellidae was resolved as the sister group to the remaining Trochidea species (BS = 98). However, phylogenetic relationships among the other four families were unstable. Turbinidae, Tegulidae, and Trochidae grouped together with weak support (BS = 66). This clade grouped with Angariidae, also without strong support (BS = 83).

Major phylogenetic relationships recovered in this study were consistent with previous studies based on Vetigastropoda mitogenomes [26,27]. However, bootstrap support values at some nodes were not high. The current matrix may not have enough phylogenetic information to resolve these nodes, so more data and research are needed for phylogenetic analyses about Vetigastropoda. *E. rumphii* was recovered as sister clade to all other vetigastropods, indicating that Pleurotomarioidea may be the most deeply diverging lineage of Vetigastropoda. This view was supported by some phylogenetic analyses based on morphology [28,29], or short molecular sequences [12,30], but first was proved by the mitochondrial genome data, as mitogenomes of Pleurotomarioidea were not included in other mitogenome-based studies. Moreover, a phylogenetic study of Vetigastropoda based on transcriptomes has been published recently [31]. Pleurotomarioidea was also recovered as sister group to all other vetigastropods in all their analyses.

### 3.5. Gene Arrangement

Nine vetigastropods of nine different families were chosen to explore Vetigastropoda mitochondrial gene arrangements (Figure 4B).

Mitochondrial gene arrangements of Vetigastropoda are mostly conserved. Five species of the order Trochida, *Granata lyrata* (Choristellidae), and *Haliotis rufescens* (Haliotidae) shared the same gene order except for the position of a very few tRNAs. The organization of their protein-coding genes was identical. As for *E. rumphii,* it showed the same gene order as *G. lyrata* and *Angaria neglecta,* and shared almost the same gene arrangement with most vetigastropods. However, *Pseudorimula* sp. (Lepetodrilidae) and *Fissurella volcano* (Fissurellidae) presented different gene arrangements. In *F. volcano*, the gene block *cytb-nad6-trnP-nad1-trnL-trnL-16S-trnV-12S-trnM-trnY-trnC-trnW-trnQ-trnG-trnE* reversed, and exchanged its position with the gene block *atp6-trnF-nad5-trnH-nad4-nad4l-trnT-trnS*. In *P.* sp, the gene block *atp8-atp6-trnF-nad5-trnH-nad4-nad4l-trnT-trnS-cytb-nad6-trnP-nad1-trnL-trnL* reversed, but did not change its position.

Stöger and Schrödl [32] compared mitogenome arrangements of different mollusca taxa. They found that most vetigastropods had the same gene order as *Octopus* and *Katharina*, so this arrangement may be plesiomorphic for gastropods. Gene arrangement of *E. rumphii* was completely consistent with the hypothetical ancestral gastropoda mitochondrial gene order, indicating its deep conservation.

Within Vetigastropoda, most species have the same gene order, except for Lepetodrilidae and Fissurellidae. Uribe [26] indicated that the high number of gene rearrangements is correlated with the high evolutionary rates of mitogenomes, and this correlation has been found in many molluscs [33,34]. This may have a negative effect on phylogenetic analyses. Therefore, mitogenomes may not be appropriate for phylogenetic reconstruction at deep nodes, compared with genome and transcriptome data.

## 4. Conclusions

The complete mitochondrial genome of *E. rumphii* was 15,795 bp in length, containing 13 PCGs, two rRNAs, and 22 tRNAs. Phylogenetic analysis based on complete mitogenomes recovered *E. rumphii* as sister group to all other vetigastropods. The arrangement of mitochondrial genes was the same as most vetigastropods as well as the inferred ancestral gastropoda. This suggested that *E. rumphii* was a deeply diverging lineage of Vetigastropoda, and its mitogenome arrangement was very conserved. This study provided a mitochondrial genome of Pleurotomarioidea, which could be helpful for understanding phylogenetic relationships and the early evolution of Vetigastropoda.

## Figures and Tables

**Table 1:** Information of species for phylogenetic analysis.

Subclass	Order	Superfamily	Family	Genus	Species	NCBI Accession
Vetigastropoda	Lepetellida	Lepetodriloidea	Lepetodrilidae	Pseudorimula	*Pseudorimula* sp.	MK404176
				Lepetodrilus	*Lepetodrilus schrolli*	KR297250
		Haliotoidea	Haliotidae	Haliotis	*Haliotis rufescens*	KX260956
					*Haliotis rubra*	AY588938
		Fissurelloidea	Fissurellidae	Fissurella	*Fissurella volcano*	NC_016953
				Diodora	*Diodora graeca*	KT207825
	Seguenziida	Seguenzioidea	Choristellidae	Granata	*Granata lyrata*	KR297249
	Trochida	Trochoidea	Tegulidae	Tegula	*Tegula rustica*	MG836833
					*Tegula nigerrima*	KX298895
			Phasianellidae	Phasianella	*Phasianella solida*	KR297251
					*Phasianella australis*	KX298888
			Trochidae	Steromphala	*Steromphala umbilicalis*	KX646541
				Monodonta	*Monodonta labio*	MK240320
				Umbonium	*Umbonium thomasi*	MH729882
			Turbinidae	Bolma	*Bolma rugosa*	KT207824
				Lunella	*Lunella correensis*	MN604179
				Astralium	*Astralium haematragum*	NC_031858
			Angariidae	Angaria	*Angaria neglecta*	KR297248
					*Angaria delphinus*	KX298893
Neritimorpha	Cycloneritida	Neritoidea	Neritidae	Clithon	*Clithon retropictum*	NC_031893
				Nerita	*Nerita albicilla*	MK516738

**Table 2:** Organization of the mitochondrial genome of *E. rumphii*.

Gene	Position		Length (bp)	Codon		Anticodon	Intergenic Region	Strand
	From	To		Start	Stop			
*cox1*	1	1536	1536	ATG	TAA		4	H
*cox2*	1562	2254	693	ATG	TAA		25	H
*tRNA-Asp*	2283	2351	69			GTC	28	H
*atp8*	2352	2516	165	ATG	TAA		0	H
*atp6*	2539	3237	699	ATG	TAG		22	H
*tRNA-Phe*	3282	3348	67			GAA	44	L
*nad5*	3359	5089	1731	ATG	TAA		10	L
*tRNA-His*	5090	5155	66			GTG	0	L
*nad4*	5162	6541	1380	ATG	TAA		6	L
*nad4l*	6535	6834	300	ATG	TAG		−7	L
*tRNA-Thr*	6842	6907	66			TGT	7	H
*tRNA-Ser^UCA^*	6907	6973	67			TGA	−1	L
*cytb*	6984	8123	1140	ATG	TAA		10	L
*nad6*	8149	8646	498	ATG	TAG		25	L
*tRNA-Pro*	8647	8714	68			TGG	0	L
*nad1*	8735	9670	936	ATG	TAG		20	L
*tRNA-Leu^UUA^*	9671	9738	68			TAA	0	L
*tRNA-Leu^CUA^*	9743	9810	68			TAG	4	L
*16S RNA*	9811	11,196	1386				0	L
*tRNA-Val*	11,197	11,265	69			TAC	0	L
*12S RNA*	11,266	12,159	894				0	L
*tRNA-Met*	12,160	12,227	68			CAT	0	L
*tRNA-Tyr*	12,267	12,333	67			GTA	39	L
*tRNA-Cys*	12,341	12,404	64			GCA	7	L
*tRNA-Trp*	12,405	12,471	67			TCA	0	L
*tRNA-Gln*	12,472	12,536	65			TTG	0	L
*tRNA-Gly*	12,552	12,621	70			TCC	15	L
*tRNA-Glu*	12,636	12,700	65			TTC	14	L
*cox3*	13,065	13,844	780	ATG	TAA		364	H
*tRNA-Lys*	13,909	13,974	66			TTT	64	H
*tRNA-Ala*	13,988	14,053	66			TGC	13	H
*tRNA-Arg*	14,054	14,121	68			TCG	0	H
*tRNA-Asn*	14,132	14,197	66			GTT	10	H
*tRNA-Ile*	14,199	14,263	65			GAT	1	H
*nad3*	14,264	14,617	354	ATG	TAA		0	H
*tRNA-Ser^AGC^*	14,624	14,693	70			GCT	6	H
*nad2*	14,694	15,791	1098	ATG	TAA		0	H

**Table 3:** Nucleotide composition of mitochondrial genomes of vetigastropods.

Species	Length (bp)	A%	T%	G%	C%	AT Skew	GC Skew	AT Content
*Entemnotrochus rumphii*	15,795	35.21	29.98	14.43	20.39	0.080225	−0.17112	65.19%
*Pseudorimula* sp.	16,682	28.76	29.79	14.47	26.98	−0.01751	−0.30181	58.55%
*Lepetodrilus schrolli*	15,579	29.39	32.79	14.90	22.93	−0.05473	−0.21236	62.18%
*Haliotis rufescens*	16,646	35.39	24.93	13.75	25.93	0.173389	−0.30719	60.32%
*Haliotis rubra*	16,907	34.57	24.55	14.18	26.70	0.169502	−0.30623	59.11%
*Fissurella volcano*	17,575	25.72	35.83	26.69	11.77	−0.16431	0.387985	61.54%
*Diodora graeca*	17,209	21.75	36.01	29.44	12.80	−0.24688	0.394139	57.76%
*Granata lyrata*	17,632	33.39	24.68	14.93	27.00	0.150029	−0.28778	58.07%
*Tegula rustica*	17,799	33.32	33.53	15.64	17.51	−0.00311	−0.05643	66.84%
*Tegula nigerrima*	17,755	33.58	33.44	15.52	17.46	0.002101	−0.05874	67.02%
*Phasianella solida*	16,698	30.41	31.63	22.13	15.83	−0.01967	0.166089	62.04%
*Phasianella australis*	18,397	30.89	34.95	20.00	14.16	−0.06159	0.171042	65.84%
*Steromphala umbilicalis*	16,277	34.74	32.44	13.09	19.72	0.034199	−0.20202	67.19%
*Monodonta labio*	16,440	31.51	27.74	13.97	26.77	0.063655	−0.31412	59.25%
*Umbonium thomasi*	15,998	34.85	29.13	13.96	22.06	0.089399	−0.22471	63.98%
*Bolma rugosa*	17,432	34.06	34.72	15.37	15.85	−0.0096	−0.01544	68.77%
*Lunella correensis*	17,308	31.03	35.30	19.93	13.74	−0.06437	0.1838	66.33%
*Astralium haematragum*	16,310	33.30	33.68	16.25	16.76	−0.00567	−0.0156	66.99%
*Angaria neglecta*	19,470	28.86	36.39	22.28	12.47	−0.11533	0.282377	65.25%
*Angaria delphinus*	19,554	28.20	36.12	22.51	13.18	−0.12309	0.261393	64.31%

## Data Availability

The mitochondrial genome of *E. rumphii* is available from GeneBank under the accession OP354269.

## References

[1] Rupert E.E., Barnes R.D. (1994). Invertebrate Zoology.

[2] Bieler R. (1992). Gastropod phylogeny and systematics. Annu. Rev. Ecol. Syst..

[3] Harasewych M.G. (2002). Pleurotomaroidean gastropods. Adv. Mar. Biol..

[4] Anseeuw P., Goto Y. (1996). The Living Pleurotomariidae: A Synopsis of the Recent Pleurotomariidae Including Color Plates of All Extant Type Specimens.

[5] Okutani T., Hasegawa K., Okutani T. (2000). Superfamily Pleurotomarioidea. Marine Mollusks in Japan.

[6] Tan T.H. (1974). A preliminary study on the anatomy of *Pleurotoniaria (Eittemnotrochus) rumphii* Schepman. Bull. Malacol. Soc. China.

[7] Woodward H. (1885). On Recent and fossil Pleurotomariae. Geol. Mag..

[8] Wenz W., Schindewolf O.H. (1938). Gastropoda. Allgemeiner Teil und Prosobranchia. Handbuch der Paläozoologie.

[9] Cox L.R. (1960). Thoughts on the classification of the Gastropoda. Proc. Malacol. Soc. Lond..

[10] Knight J.B., Cox L.R., Keen A.M., Batten R.L., Yochelson E.L., Robertson R., Moore R.C. (1960). Gastropoda, Systematic Descriptions. Treatise of Invertebrate Paleontology. I—Mollusca 1.

[11] Graham A., Trueman E.R., Clarke M.R. (1985). Evolution within the Gastropoda: Prosobranchia. The Mollusca. Volume 10—Evolution.

[12] Harasewych M.G., Adamkewicz S.L., Blake J.A., Saudek D., Spriggs T., Bult C.J. (1997). Phylogeny and relationships of pleurotomariid gastropods (Mollusca: Gastropoda): An assessment based on partial 18S rDNA and cytochrome coxidase I sequences. Mol. Mar. Biol. Biotechnol..

[13] Boore J.L. (1999). Animal mitochondrial genomes. Nucleic Acids Res..

[14] Bernt M., Braband A., Middendorf M., Misof B., Rota-Stabelli O., Stadler P.F. (2013). Bioinformatics methods for the comparative analysis of metazoan mitochondrial genome sequences. Mol. Phylogenet. Evol..

[15] Kern E.M.A., Kim T., Park J.K. (2020). The Mitochondrial Genome in Nematode Phylogenetics. Front. Ecol. Evol..

[16] Ravi K.P., Mukesh J. (2012). NGS QC Toolkit: A Toolkit for Quality Control of Next Generation Sequencing Data. PLoS ONE.

[17] Bankevich A., Nurk S., Antipov D., Gurevich A.A., Dvorkin M., Kulikov A.S., Lesin V.M., Nikolenko S.I., Pham S., Prjibelski A.D. (2012). SPAdes: A new genome assembly algorithm and its applications to single-cell sequencing. J. Comput. Biol..

[18] Zhang D., Gao F., Jakovlić I., Zou H., Zhang J., Li W.X., Wang G.T. (2020). PhyloSuite: An integrated and scalable desktop platform for streamlined molecular sequence data management and evolutionary phylogenetics studies. Mol. Ecol. Resour..

[19] Lowe T.M., Chan P.P. (2016). tRNAscan-SE On-line: Search and Contextual Analysis of Transfer RNA Genes. Nucleic Acids Res..

[20] Lowe T.M., Chan P.P. (1997). tRNAscan-SE: A program for improved detection of transfer RNA genes in genomic sequence. Nucleic Acids Res..

[21] Katoh K., Standley D.M. (2013). MAFFT multiple sequence alignment software version 7: Improvements in performance and usability. Mol. Biol. Evol..

[22] Talavera G., Castresana J. (2007). Improvement of phylogenies after removing divergent and ambiguously aligned blocks from protein sequence alignments. Syst. Biol..

[23] Kalyaanamoorthy S., Minh B.Q., Wong T.K.F., Haeseler A., Jermiin L.S. (2017). ModelFinder: Fast model selection for accurate phylogenetic estimates. Nat. Methods.

[24] Minh B.Q., Schmidt H.A., Chernomor O., Schrempf D., Woodhams M.D., Haeseler A., Lanfear R. (2020). IQ-TREE 2: New Models and Efficient Methods for Phylogenetic Inference in the Genomic Era. Mol. Biol. Evol..

[25] Garey J.R., Wolstenholme D.R. (1989). Platyhelminth mitochondrial DNA: Evidence for early evolutionary origin of a tRNA AGN that contains a dihydrouridine arm replacement loop, and of serine-specifying AGA and AGG codons. J. Mol. Evol..

[26] Uribe J.E., Kano Y., Templado J., Zardoya R. (2016). Mitogenomics of Vetigastropoda: Insights into the evolution of pallial symmetry. Zool. Scr..

[27] Lee H., Samadi S., Puillandre N., Tsai M.H., Dai C.F., Chen W.J. (2016). Eight new mitogenomes for exploring the phylogeny and classification of Vetigastropoda. J. Molluscan Stud..

[28] Boss K.J., Parker S.P. (1982). Mollusca. Synopsis and Classification of Living Organisms.

[29] Vaught K.C. (1989). A Classification of Living Mollusca.

[30] Yoon S.H., Kim W. (2005). Phylogenetic Relationships Among Six Vetigastropod Subgroups (Mollusca, Gastropoda) Based on 18S rDNA Sequences. Mol. Cells.

[31] Tauana J.C., James D.R., Gonzalo G. (2022). Investigating Sources of Conflict in Deep Phylogenomics of Vetigastropod Snails. Syst. Biol..

[32] Stoger I., Schrodl M. (2013). Mitogenomics does not resolve deep molluscan relationships (yet?). Mol. Phylogenet. Evol..

[33] Rawlings T., MacInnis M., Bieler R., Boore J., Collins T. (2010). Sessile snails, dynamic genomes: Gene rearrangements within the mitochondrial genome of a family of caenogastropod molluscs. BMC Genom..

[34] Schrodl M., Stoger I. (2014). A review on deep molluscan phylogeny: Old markers, integrative approaches, persistent problems. J. Nat. Hist..

